# Typhoid Fever

**Published:** 1895-11-30

**Authors:** 


### Progress in Medicine.

### TYPHOID FEVER.

**Ætiology.**—T. Priestly,[1] of Leicester, believes that besides infection through water or milk, typhoid may possibly spread directly; at least, single cases in houses in Leicester, where the sewage is retained in pails, are often followed by others, though whether by atmospheric infection or personal contact is not clear. The presence of active bacilli in the urine may be a common and neglected source of infection, according to D. Semple,[2] who found them in six out of seven cases which he examined. This may explain, too, the frequent infection of nurses, for their precautions too often extend only to contamination by fæces, which more rarely contain the germs. He suggests that the diagnosis of typhoid may thus be aided by testing the urine, and suggests that the disease is a true septicæmia, not an "intoxication process" caused by germs living in the intestines. Indeed, it has been shown that they are rapidly killed in the intestines by the B. coli; and possibly the ulceration is the work of the latter microbe acting on the adenoid tissue inflamed by the typhoid toxin, or otherwise may be due, like the skin spots, to bacilli which have lodged in a capillary. The constant occurrence of bacilli in the urine is rather in favour of the septicæmic theory.[2] The extraordinary mortality from typhoid in the troops abroad, where it is rare among the natives, is explained by W. L. Chester[3] on the theory that in "pre-sanitary days" every child takes it and becomes protected. Thus the natives of Toulon are proof against it, while the mortality among strangers is very high. The children there, too, suffer from what is termed a mucous fever, which is rarely fatal, and is probably mild typhoid. A similar immunity has been found to some extent in the Trent Valley. The incidence of typhoid for British troops in India has been 20 per 1,000 during the last decade, in place of 14·7 for the previous one, and the death-rate 5·29, in place of 4·13; while among the natives, the Goorkhas and the higher-class Mahommedans alone, both of whom are flesh-eaters, are materially affected.[4] At home, the officer of health for Brighton found that 40 per cent. of the typhoid there in 1894 was due to infected oysters and other shell fish.[5] The general scare on the subject has lessened the consumption by four and a half millions of oysters in three months; but however this is to be regretted, the public require a wholesome fish, and there is no reason why this should not be supplied.[6] A milk epidemic, attacking eighty persons, occurred at Great Harwood,[7] and another at Stamford, U.S.A.[8]; but S. D. Rowland[9] reports that, apparently, English cheese and butter are not carriers of infection, for the germs quickly died when planted on these substances, though Hankin[10] found them in the "dahi" made from Indian milk. To the enterprise and energy of Ernest Hart we owe a long historical account of the various epidemics produced by polluted water from 1863 to 1893 in the United Kingdom. He summarises the effects of 206 of those outbreaks. They are due to polluted wells, leaky sewers, contamination of water-works by infected labourers, suction of air and sewage into the mains, the action of frost, polluted subsoil, and the use, though filtered, of infected river water. He is inclined to regard the soil as the natural habitat of the typhoid germ, but believes it is rarely conveyed to man except through water which has been contaminated by fæcal matter. In conclusion, he points out the great gain, even from a financial point of view, of stamping out typhoid by a thorough purification of the water supply of the entire country. Against this vast amount of evidence for the frequency of water-borne typhoid there is little to be said; but J. C. Battersby[11] remarks that certain Indian epidemics, such as that in the Chitral Relief force, could not have been due to it, but rather to the flies which hover about the food and excrements alike of every house and camp. In this campaign the water and camping grounds appeared quite above suspicion, and the disease was apparently carried with the troops.

Ram Kishen[12] states that the disease is far from uncommon among natives, but that the majority of the population undergo it during infancy, and in the adults the attacks are mild. Spots and diarrhœa are rarely seen, but even in ancient Persian works the existence of the disease is clearly noticed. The points of difference between the typhoid and colon bacilli have been found by Klein[13] to remain unaltered when these germs are grown for long periods outside the body. Inoculation of calves and a monkey with typhoid matter was successful though difficult. Kanthack[14] considers that the presence of B. coli is not a proof of fæcal contamination, for it occurs in dust, earth, frozen mutton, and in many other localities least likely to be thus infected. The toxins produced in typhoid are little known—the latest account of them is given by Soltau Fenwick and Bokenham.[15] They found in the spleens of persons dying in the third week of the disease from two to four grains of an albumose, which causes pyrexia, loss of appetite, and emaciation when injected into animals. An alkaloid and fatty acid found with the albumose had no toxic properties.

**Complications.**—If we now turn to consider some of the complications of typhoid, it is useful to notice that the end of the disease itself is shown by the concurrence of hypothermia slow pulse, and polyuria, but besides complications common to all stages we may now occasionally find osteal manifestations, cholelithiasis, subcutaneous abscesses, mental aberration, and various forms of neuritis. Thus Osler[16] records four cases of general peripheral neuritis and five of local neuritis. Two of the latter recovered completely. W. Sinkler mentioned one of paraplegia, and C. S. Potts one of myositis, while cases of progressive muscular atrophy of the peroneal type are known, and Gowers[17] regards typhoid as specially fruitful in polio myelitis. We need not pause over those cases abroad where strange symptoms are due to the concurrence of malaria with typhoid, except to repeat that no hybrid or true mixture takes place;[18] but there are many others of interest, such as renal complications. If these are not due to the pyrexia, but come on at the end of the third week, the prognosis is serious, and, according to Lecoq, the mortality is 66 per cent.[19] Cystitis and pyelitis[20] may appear even in early stages of the disease, and even acetonæmia, as in a case reported by A. Todd.[21] Thrombosis of both femoral veins occurred in a patient under Dr. Cooper,[22] and a fatal termination followed. Hanot also recently recorded among heart lesions a constant *bruit de galop*, especially in the region of the sternum, with signs of dilatation of the right heart, which had hitherto been unnoticed.[23]

**Treatment.**—Berg[24] gives an analysis of 1,626 cases treated at Leipzig by the expectant method. The mortality was 12·7 per cent., varying in different years from 7 to 18 per cent. C. A. Ray,[25] in discussing a group of 81 cases with only a mortality of 3⅝ on a mixed treatment with an initial dose of calomel, takes occasion to show the danger of acetanilid and antifebrin. One of his deaths was due to the former. Woodbridge[26] has advocated cathartic remedies in children, and Thistle,[27] of Toronto, reports 172 cases with a mortality of only 3 per cent., treated by active continued purgation and copious draughts of water. He had no case of perforation or death from toxæmia, and only eight cases of hæmorrhage, two being among the fatal ones. He argues that purgation tends to remove the caustic toxins and prevent perforation. Temperature and pulse rapidly improved under his method, and diarrhœa was rarely seen. The paper is certainly a suggestive one in many ways. W. Osler,[28] on the other hand, in five years' experience of the cold bath treatment, claimed a death-rate of 6·3 per cent., and spoke against excessive use of drugs. W. S. Barker[29] in 35 cases had no death under the bath treatment, for which he employed the common tin domestic bath in the case of private patients, but it is not clear how the patients were lifted in and out. Le Gendre would use the cold bath where the temperature is 104 and the morning remission is slight, but when it is only over 102 he advises the progressively-cooled bath. He carefully disinfects the mouth and nose, and washes out the colon daily with a quart of boric or naphthol solution. He gives calomel at the beginning, and afterwards salines every third day, except hæmorrhage or peritonitis is present. Benzonaphthol and quinine are also given largely in repeated doses during the day. Altogether his patients cannot complain of want of due antisepsis.[30] Among other methods we may notice that of W. Ewart,[31] who recommends initial doses of calomel or castor oil, followed by a mixture of the perchlorides of iron and mercury every four hours throughout the disease, with the special view of aiding the liver as well as with other objects. He gives a fair amount of carbohydrates in the diet, such as arrowroot for the first fortnight, and returns to them early during convalescence. The experience of Zinn[32] for the last five years is in favour of the bath treatment, with or without quinine. The treatment by Yeo's chlorine mixture is the subject of papers by W. G. Myers[33] and R. W. Wilcox. The former used also the initial calomel purge, and found that in some cases the temperature was kept down permanently by the chlorine, and the patient's strength and mental condition were superior even to those under the bath treatment. Hæmorrhages occurred in three cases. Wilcox[34] praises it for similar results, and believes the disease is shortened in duration. He reserves its use for cases seen late, where ulceration and systemic infection is marked. In early cases he employs salol and other compounds, and gives baths when required. Serum treatment has been attempted again by Klemperer and Levy,[35] who immunised (?) a dog and injected 20 c.c. of its serum every evening for three days in five cases of typhoid. The temperature became normal about the end of the second week. So far the injections appear harmless, and the effect would seem to be merely to hasten the end of the fever. Rumpf[36] found that sterilised cultures of B. pyocyaneus in 60 to 80 per cent. of the cases reduced the term of pyrexia, probably by exciting the protective powers of the organism, but the method is not yet worked out for practical purposes. Injections of thymus extract have been tried by Lambert,[37] Northrup, and Brannan. Lambert employed as much as 6 or 7 c.c. for a dose. In some cases no effect was produced; in others there was evidence of improvement, but the results and indications are not clear.

Guiacol is recommended by various writers. McCormick[38] speaks of it as superior to cold baths, easy to apply, and quite safe if used with ordinary care, and without harmful effects. A. P. Hull[39] employed it both externally and internally, and agrees with McCormick as to its effects, adding that it inhibits the action of the B. coli. T. S. Carpenter,[40] while recording several cases of temporary collapse, found it invaluable in two epidemics. He usually employs 10 to 20 drops externally, and the painting may be repeated after two hours with a smaller amount if no effect is produced. The perspiration usually occurs in thirty minutes, and in extreme cases the application of cold sponges may be employed in addition, or subsequently to maintain the effect.

[1] B. M. J., August 31. [2] Lancet, July 27. [3] Lancet, August 31. [4] Lancet, May 18. [5] B. M. J., June 8. [6] B. M. J., May 25. [7] Lancet, May 25. [8] Med. Rec., May 4. [9] B. M. J.. June 22. [10] B. M. J., June 15 to August 17. [11] B. M. J., August 10. [12] Int. Med. Rev., July. [13] B. M. J., Sept. 28. [14] B. M. J., July 27. [15] B. M. J., April 13. [16] Med. Week, April 19. [17] T. Nerv. and Ment. D., May. [18] U.S. Med. Bulletin, April 1. [19] B. M. J., August 31. [20] B. M. J., April 13. [21] B. M. J., May 11. [22] B. M. J., May 18. [23] Med. Week, April 26. [24] Amer. Journ. Med. Sci., August. [25] Med. Rec., May 8. [26] Med. Rec., May 11. [27] Med. Rec., Sept. 14. [28] Med. Rec., Sept. 14. [29] Ther. Gazette, August 15. [30] Amer. Journ. Med. Sci., June. [31] Med. Press (see p. 505), 1895. [32] B. M. J., June 29. [33] Amer. Journ. Med. Sci., June. [34] Amer. Journ. Med. Sci., Sept. [35] Med. Rec., August 17. [36] Amer. Journ. Med. Sci., August. [37] B. M. J., April 27. [38] Ther. Gazette, April 15. [39] Ther. Gazette, August 15. [40] Ther. Gazette, June 15.

